# Connecting Recreational Service to Visitor’s Well-Being: A Case Study in Qianjiangyuan National Park

**DOI:** 10.3390/ijerph191811366

**Published:** 2022-09-09

**Authors:** Wenjia Zhou, Jun Cai, Kai Chen

**Affiliations:** 1 School of Economics and Management, Beijing Forestry University, Beijing 100083, China; 2 School of Landscape Architecture, Beijing Forestry University, Beijing 100083, China

**Keywords:** ecosystem recreational services, ecosystem service flow, tourist’s well-being, Qianjiangyuan National Park

## Abstract

Natural ecosystems provide service value to people in the region; especially in some nature reserves and national parks, the ecosystem recreational service function is more significant. It is worth paying attention to whether the recreational service function of this ecosystem can serve tourists outside the region and improve their well-being. Taking Qianjiangyuan National Park in China as the research site, based on the Spatially Explicit Ecosystem Services Comprehensive Assessment Model (ESTIMAP), we used the ecosystem services mapping tool to propose a comprehensive assessment framework for ecosystem recreational services and to explore the relationship between recreational services and the well-being of tourists. As a result, we identified the spatial distribution of the supply and demand flow paths of ecosystem recreational services and estimated that the actual flow of ecosystem recreational services was about 12.1 persons/km^2^ per year. In terms of personal well-being, ecosystem recreational services have a significant positive impact on tourists’ well-being. The service value is concentrated in amusement places and recreational activities, and dynamic recreational activities have a greater impact on tourists. The research results suggest that to improve the well-being of tourists and the value of ecosystem recreational services, national parks and nature reserves should improve accessibility and the participation of tourists in recreational activities.

## 1. Introduction

The concept of ecosystem services describes the benefits people obtain from ecosystems. It mainly includes four kinds of services: (1) provisioning services, such as providing food, water and other resources; (2) regulating services, such as mitigating extreme climate impacts; (3) cultural services, such as providing recreational places; (4) support services, such as ecosystem nutrient cycling [1]. There is much debate about the relationship between ecosystem services and well-being. Some environmentalists believe that ecological degradation will lead to a decline in the well-being of people who depend on ecosystem services, a hypothesis based on unsustainable rates of resource consumption that have severely affected human well-being in the past [2]. The Millennium Ecosystem Assessment, on the other hand, reflects the close feedback between ecosystem services and human well-being, showing that human well-being has increased despite a significant decline in most ecosystem services globally.

In fact, assessing the value of ecosystem services can contribute to human well-being. Ecosystem service function (ESF) is not viewed as a natural phenomenon like biological sciences but as the potential of ecosystem services (ESP), thus making it amenable to economic calculation [3,4]. The value of ecosystem services lies in how to convert the ecosystem service functions of biophysical attributes into ecosystem service potentials, and these potentials must be mobilized to provide ecosystem services. Once the potential is allocated, the ecosystem benefits from these services may or may not be commercialized [5,6]. Raudsepp-Hearne et al. (2010) pointed out that a better understanding of how human well-being depends on ecosystems requires research that integrates human well-being, agriculture, technology and ecosystem services. This mainly includes four aspects: human well-being improved by ecosystem services, synergies and trade-offs of ecosystem services, technologies to enhance ecosystem services, and forecasting of ecosystem services [7], and the role of ecosystem cultural services is particularly significant.

Ecosystem cultural service is a part of ecosystem services that is characterized as subjective, intangible and difficult to quantify in monetary terms. This may generate a multitude of intangible and non-market benefits (such as social cohesion), which in turn can hold or give it different values (such as morality, religion, aesthetics, etc.) [8,9]. Schröter et al. (2014) conducted a spatial analysis of Telemark County in southern Norway using a spatial model and proposed nine indicators of ecological services, including moose hunting, sheep grazing, logging, carbon sequestration and forest sequestration, avalanche prevention, residential facilities, leisure excursions and the presence of undisturbed areas [10]. In addition, cultural service values vary over time and societies, differing in their public accessibility and the degree of development of human–ecosystem interactions that occur as cities grow. Over time, there is a shift from intrinsic interpersonal values (spiritual, cultural heritage) to instrumental interpersonal values (entertainment, education) [11].

Since 2019, under the influence of the COVID-19 epidemic, people’s physical and mental health has been positively correlated with entering the outdoor landscape [12]. Especially in urbanized landscapes, the importance of well-being brought by recreational services may exceed other ecosystem services [13], and the value of ecosystem cultural services provided by ecosystems such as parks and green spaces in cities is in increasingly important in modern human life [14,15,16]. Ferretti-Gallon (2021) proposed that national parks were widely regarded as the ultimate opportunity to support tourism while preserving ecosystem integrity [17]. The national parks trigger strategic shifts and new approaches to destination development that challenge previous strategies [18]. For tourism destinations, digital and mobile marketing, infrastructure, branding, quality, accessibility and informational factors are important means of promoting tourism [19]. Through the investigation of the national parks in southern Utah, Templeton (2021) researched the fact that, under the influence of COVID-19, national parks were facing both management challenges and market opportunities. New Internet technologies such as social media play an important role in park management and marketing [20]. Florido-Benítez (2022) pointed out that, during the epidemic, international mobile marketing was an important tool to identify, predict and meet customer needs [21].

Most studies on the valuation of ecosystem recreational services use value assessment methods, such as the Tourism Cost Method (TCM) and the Conditional Value Method (CVM), to quantify the value of recreational services and measure their actual service levels. Larson (2015) studied residential landscape ecosystem services for residents of six USA cities, emphasizing that the cultural services of ecosystems are crucial, especially as they correspond with human-centered values, including aesthetics, low maintenance and personal enjoyment [22]. Agimass (2018) used questionnaire spatial data analysis to evaluate the recreational value provided by the forest ecosystem in Denmark based on attributes such as forest area, density, tree species composition, historical sites and terrain differences [23]. Using methods such as spatial analysis and service mobility analysis, participatory mapping, and other technologies such as remote sensing satellite maps and GIS, the spatial flow service path characteristics of ecosystem services can be analyzed and modeled for land types, accessibility and other characteristics, so as to explore their spatial patterns and key points in order to facilitate the identification of types and areas of ecosystem service trade-off synergy. For the study of ecosystem recreational service flow, Zulian (2014) proposed the ESTIMAP (Ecosystem Service Mapping Tool) model, which differs from land use cover-type maps in that it adds other spatial information, originally used in the European Union to support land use policies, including air quality regulation of ecosystem services, coastal protection and crop pollination, and recreational functions [24]. Vallecillo (2019) builds on this framework and uses it to assess the recreational potential, demand and service flows of natural ecosystems in the EU [25].

A common hypothesis is that ecosystem services affect the well-being of Indigenous peoples within ecosystems [26]. However, for tourists who enter the ecosystem, whether they can enjoy the benefits of ecosystem services and what impact the value of ecosystem recreational services will have on the well-being of tourists are issues that deserve attention. In tourism development, ecosystem services and tourism-oriented urbanization interact and reinforce each other. Ecosystem services are the entry point for objective and quantitative assessment of well-being, and understanding the flow of ecosystem recreational services and the capacity of ecosystems to generate these services is critical to understanding the sustainability of ecosystem recreational use and improving visitor well-being. Among the first pilot national park systems in China, Qianjiangyuan National Park is the only one located in the Yangtze River Delta, a densely populated and economically developed region. The ecosystem of the Qianjiangyuan area is intact and typical of subtropical evergreen, broad-leaved forest zonal vegetation. Therefore, based on RS and GIS techniques, we propose a framework for comprehensively evaluating ecosystem recreational services from both macro and micro perspectives. At the macro level, we assess the value of ecosystem recreational services while showing spatial distribution patterns. Furthermore, at the micro level, we establish scales to measure visitor well-being and to analyze the impact of ecosystem recreational services on human well-being. It is of great significance to improve the recreational service function of the ecosystem and to enhance human well-being.

## 2. Materials and Methods

### 2.1. Research Area

**Figure 1:**
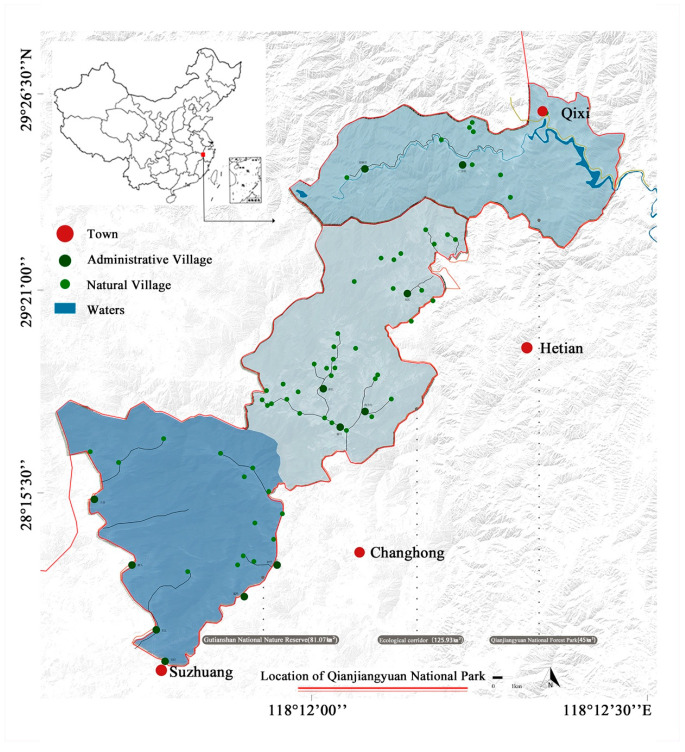
Location of Qianjiangyuan National Park.

The Qiantang River is the largest river in Zhejiang Province, China, and the Qianjiangyuan National Park is where the source of the river is located. The geographic coordinates are between 118°03′~118°40′ E and 29°10′~29°30′ N, as shown in Figure 1. It includes several nature reserves with large areas of primitive broad-leaved forests in the middle subtropics, which are important ecological barriers and water-supporting areas in China and have high ecological value. The river valleys, forests and wetlands in the park nurture rich and diverse biological resources and are an important biological gene pool. The area is a centralized distribution area for *Syrmaticus ellioti*, *Muntiacus crinifrons*, *Panthera pardus* and *Neofelis nebulosa*; all four types of wildlife are rare and endangered species in the world.

Two field studies were conducted on Qianjiangyuan National Park, in October 2019 and October 2020, for which 128 and 276 questionnaires were obtained, respectively. After the questionnaires were collected, the data were compiled and counted, and SPSS 25.0 (IBM, Armonk, NY, USA) was used to enter the questionnaire data and conduct statistical analysis. The questionnaire was designed mainly for tourists, aiming to grasp the demographic characteristics and basic recreational characteristics of tourists, investigate tourists’ perceptions of ecosystem services and tourists’ well-being, and understand tourists’ actual expenses and willingness to pay for ecosystem recreational value during the trip, so as to provide basic data for the study of the recreational value of Qianjiangyuan National Park.

### 2.2. Ecosystem Recreational Service Value Assessment

**Figure 2:**
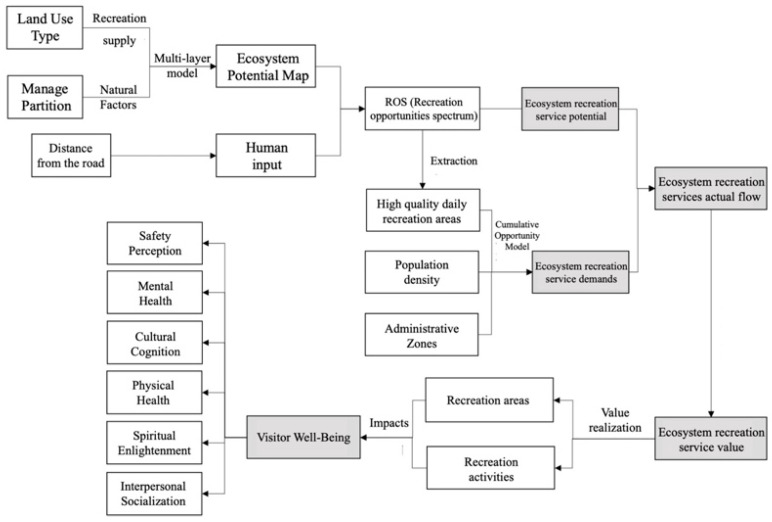
Integrated Assessment Framework for Ecosystem Recreational Services.

The value of recreational services in the ecosystem of Qianjiangyuan National Park was assessed, including the spatial distribution of recreational service flows and the monetary value of recreational services, where recreational service flows included the supply (potential) of recreational services (Section 2.2.1), demand (Section 2.2.2) and spatial distribution of actual flows between the two. On this basis, the value of recreational services in the ecosystem of Qianjiangyuan National Park was evaluated, and the actual flow as converted into a monetary value measurement through the travel cost method. The perceived and actual embodiment of the value of recreational services by tourists was analyzed by questionnaire method, and the actual benefit transformation of the value of recreational services and the impact on the well-being of tourists were explored (Figure 2).

#### 2.2.1. Ecosystem Recreational Service Potential

Quantifying the value of ecosystem recreational services relies on spatially explicit models developed from biophysical assessments of ecosystems, which are based on ecosystem service mapping tools [27]. The spatially explicit information used to measure ecosystem services includes the potential, demand and actual flows of ecosystem services [28,29,30]. The analysis of the potential of ecosystem recreational services is based primarily on their natural properties. People engage in recreational activities in nature that encompass passive or active observation, experience or enjoyment of the biophysical characteristics or qualities of ecosystems, including hiking in the forest, walking in nearby rivers, biking through mountain trails, picnicking or observing plants [31,32]. Thus, areas with more recreational opportunities facilitate more recreational activities and thus have greater potential for recreational services.

According to the ESTIMAP map tool proposed by Zulian et al., the recreation potential is mainly measured based on the type of land use and accessibility [33]. Through literature combing, the recreation potential scale for land use types in Qianjiangyuan National Park was established, and 17 experts were consulted using the Delphi method to score the recreation potential of different land types with a score out of 10; the average assigned values of recreation potential for land types in Qianjiangyuan National Park were obtained (Appendix A).

#### 2.2.2. Demand for Ecosystem Recreational Services

According to the survey of local visiting tourists, Zhejiang Province was the main source of visitors to Qianjiangyuan National Park, with about 82.25% of visitors coming from the provincial area. Therefore, the demand for ecosystem recreational services in Qianjiangyuan National Park was analyzed by taking Zhejiang Province as the main source of visitors. The measurement of the demand for ecosystem recreational services in the source areas of Zhejiang Province was mainly based on the distance cost of each region to reach the national park, which is the accessibility of visitors in each region. Traffic accessibility was calculated by the following equation:

*A_i_* = min (*M_j_T_ij_*) (1)

**Remark** **1.** *T_ij_ is the passage time of point i to reach point j by the shortest route in the traffic network; M_j_ is the weight of point j, and the value is 1 if only traffic accessibility is studied; A_i_ is the accessibility of point i in the study area; i and j are two points in the region.*

By spatially analyzing the distance cost, the distance cost to reach the Qianjiangyuan National Park in Zhejiang Province was calculated (Appendix B). In general, the demand for ecosystem recreational services was basically consistent with the accessibility of each region in the province to the national park and was also influenced by the level of local regional economic development. Regions closer to Qianjiangyuan National Park have lower distance and time costs, higher accessibility and higher demand for ecosystem recreational services. Regions with more developed economies have higher per capita income levels and relatively higher demand. Qianjiangyuan National Park mainly serves the proximity market, which is a user market for short, scheduled recreational activities with high accessibility, and the national park promotes the daily recreation of the surrounding proximity market. Moreover, as a pilot national park, Qianjiangyuan National Park has become much more well-known and has attracted many user markets outside the region, which are relatively high in economic level and have higher recreation demand.

### 2.3. Actual Flow of Ecosystem Recreational Services

In order to explore the actual flow of ecosystem recreational services in the Qianjiangyuan National Park, it was necessary to quantify the number of potential visits generated in areas of different distances. The migration rate function represents the probability of potential visits as a function of distance travelled. Referring to Geurs and Ritsema (2008) for the log-logistic equations of visits to places [34] and modeling the number of daily visits based on the spatial distribution of the population, the migration rate function was applied in the following manner:

(2) $$N=\sum_{i=1}^{n}\frac{\left(1+k_{i}\right)}{\left(k_{i}+e^{\left(-a_{i}\times Pop_{i}\right)}\right)}$$

**Remark** **2.** *N is the number of visits to recreation per week. Pop_i_ represents the number of populations in distance buffer i, n is the number of buffers at different distances, and k_i_ and a_i_ are the parameters of interest.*

**Figure 3:**
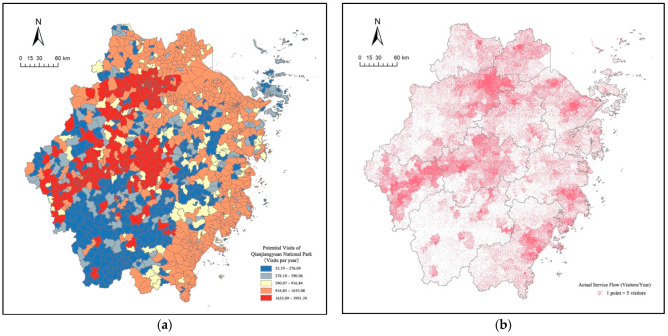
(**a**) The potential visits to Qianjiangyuan National Park in Zhejiang Province; (**b**) The actual service flow of recreational ecosystem services of Qianjiangyuan National Park.

According to the migration rate function, the number of regional populations can be moved to high-quality recreational areas by relying on traffic movement. The potential number of recreational trips within different administrative units was calculated using the number of intra-regional population in different distance intervals in Qianjiangyuan National Park. The data were labeled according to point density, with each point representing 5 visits, to obtain the actual flow of recreational services in the Qianjiangyuan National Park ecosystem (Figure 3). The actual flow of the ecosystem recreational service in Qianjiangyuan National Park in Zhejiang Province was about 1.27 million visits, according to the summary calculation of the actual flow of each region. The total area of Zhejiang Province is 105,500 square kilometers, and the actual flow of ecosystem recreational services was calculated to be about 12.1 visits/km^2^ per year on average.

### 2.4. Measurement of Visitor Well-Being

We measured the well-being of 276 visitors to Qianjiangyuan National Park through a questionnaire survey. Among the respondents, 61.23% were male, and 38.77% were female. The number of middle-aged tourists, aged 46–55 and 36–45, was the largest, respectively accounting for 25.36% and 20.29%, and the young tourists aged 18–25 and 26–35 reached 16.67% and 21.38%. In total, 82.97% of the tourists visited Qianjiangyuan National Park for the first time and stayed for about one to two days.

The sample’s sampling suitability for factor analysis was very high and reached a significant level by KMO and Bartlett’s spherical test. Therefore, factor analysis was conducted on 24 question items related to well-being. The well-being factor consisted of six elements: safety perception, mental health, cultural cognition, physical health, spiritual enlightenment and interpersonal socialization [35]. The specific content scores in each well-being factor are shown in Table 1.

## 3. Results

### 3.1. Spatial Distribution of Ecosystem Recreational Service Value

#### 3.1.1. Spatial Distribution of Recreational Potential

Based on the land use type assignment, we studied the spatial distribution of the recreational service potential of the national park ecosystem in combination with the national park management districts. According to the recreational service potential scores of different areas, Qianjiangyuan National Park was classified into four recreation potential areas, which were high recreation potential (10.291–12.000), middle recreation potential (8.711–10.290), general recreation potential (7.001–8.710) and low recreation potential (5.240–7.000) (Figure 3).

The spatial distribution of the recreation potential shows that the highest potential areas were mainly distributed in a point-and-line pattern, with discontinuous areas and characteristics of distribution along river basins and reservoir waters. The high potential areas were mainly distributed in a surface pattern with a relatively large area covering most of the traditional use areas of the countryside. In the northeast of the national park, Majin Creek and its tributaries flow through, which is the origin of the Qiantang River system and where the Qixi Reservoir was built. The Kukeng Reservoir was also built in the central area. At the same time, Gutian Mountain in the southwest is a national nature reserve with certain recreational utilization areas, superior conditions of natural environment and flora and fauna resources, and the construction of tourism service reception facilities; this area also has high potential for recreational services.

#### 3.1.2. Ecosystem Recreational Services Based on ROS

Accessibility is another important reference indicator for recreational opportunities. Areas close to transport roads have relatively better infrastructure and higher accessibility. As a result, their potential to support daily recreational and leisure activities is also higher. Spatial analysis of built infrastructure, such as transportation roads, is particularly important for assessing recreation potential. The natural recreation potential of the ecosystem itself and the accessibility of the roads together determined the recreation opportunity spectrum of the area.

**Figure 4:**
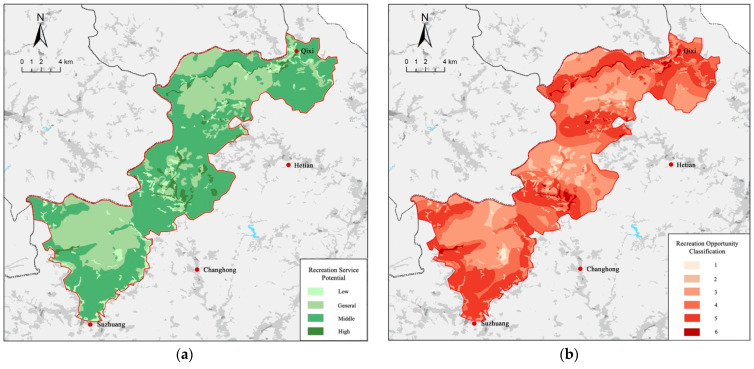
(**a**) The potential distribution of recreational services in Qianjiangyuan National Park; (**b**) Recreation Opportunity Spectrum of Qianjiangyuan National Park.

Considering the high population density and dense road network in China, the four levels adjacent to the road and the four areas with different recreation potential were cross-tabulated to obtain the Recreation Opportunity Spectrum (ROS) of Qianjiangyuan National Park by combining the road network level and density of the national park. A spatial model with six categories of recreation opportunities was obtained, reflecting the different levels of provision potential for national park recreational activity and road proximity for accessibility (Figure 4).

The analysis shows that the area with high service potential was mainly distributed along roads and rivers, covering most of the rural areas. This area is flatter, with better infrastructure construction and more convenient traffic compared to mountainous areas. The recreation potential of rural areas should be explored. During the peak season period, the forest has a certain upper limit of tourist-carrying capacity, and rural tourism in the surrounding rural areas has a high potential for the development of the national park.

### 3.2. Distribution of Actual Flow of Ecosystem Recreational Services

First, the actual recreational service flow of the ecosystem was concentrated in the local area of Quzhou City. Most of the visitors to the national park came from Quzhou city. Local residents went to the national park for recreational activities such as daily leisure, fitness and entertainment. They can reach Qianjiangyuan National Park within 2 h by car, which is less time-consuming and has high accessibility. Therefore, the potential for visits for recreation is high.

Second, the distribution of the actual ecosystem recreational service flow in Zhejiang Province was mainly concentrated in urban areas with a developed economy, high population density and close proximity to national parks. For example, Hangzhou and Ningbo have a high population density, a high level of economic development and high per capita economic income, and residents’ demand for recreation is relatively large; thus, the potential for visits for recreation is high. Meanwhile, Hangzhou, Jinhua and other cities are adjacent to Quzhou City and have convenient transportation to reach the national park, so their potential for recreational visits is also high. Although Lishui is close to the national park, its economic development level and recreational demand are relatively low, so its potential for recreational visits is also low.

### 3.3. Ecosystem Recreational Service Value Assessment

Applying the zonal tourism cost model, the variables involved in the model mainly include tourism rates, tourism costs and income levels of tourists in different regions. We established a regression model using the weighted least-squares method, with travel rate as the dependent variable and travel cost and income level as independent variables. Using the population and disposable income of each source area, a functional relationship between travel costs and actual tourism demand can be derived for each division.

It was calculated that the recreational value of the national park was higher in Hangzhou and Ningbo. The high recreational value was mainly distributed in areas with high accessibility and a high economic development level, which are close to the national park. This is consistent with the spatial distribution pattern of the actual flow of recreational services in the ecosystem of Qianjiangyuan National Park. Meanwhile, the total recreational value of Qianjiangyuan National Park is about 1.802 billion RMB, and each person can enjoy about 1456.62 RMB of recreational value per trip on average.

The high recreational value of Qianjiangyuan National Park is due to the fact that the national park is far away from the urban area, with superior natural environmental conditions and a high level of recreation potential. The geographical location is located in the eastern coastal area of China, and the surrounding areas are economically developed. Tourists have less leisure time and a high time cost, and the level of consumer surplus and tourism consumption is also high. Therefore, the recreational value is also higher.

### 3.4. The Influence of Ecosystem Recreational Services on Visitor Well-Being

As shown in Table 1, the highest scores of 4.75 were obtained for “the air in Qianjiangyuan is fresh and pollution-free” and “the water in Qianjiangyuan is clear and of good quality”, indicating that the good natural environment of Qianjiangyuan was highly recognized by tourists. Secondly, the well-being scores for physical health, mental health and spiritual enlightenment were also high. This indicates that Qianjiangyuan National Park provides visitors with a place for fitness and leisure, and the recreational activities in it are relaxing and enjoyable. In contrast, the two well-being scores for interpersonal socialization and cultural cognition were lower, which was analyzed to be related to the obvious natural resources and environmental characteristics of Qianjiangyuan, but less historical and cultural embodiment.

The value of ecosystem recreational services is reflected in both recreational sites and recreational activities [36]. In the above section, the spectrum of recreation opportunities provided by national parks was constructed, and the spatial distribution of areas with high recreation potential was explored. Visitor well-being was classified into six dimensions: perceived safety, psychological well-being, cultural awareness, physical well-being, spiritual enlightenment and interpersonal socialization. The relationship between ecosystem recreational services and visitor well-being was examined by the K–S normality test and Pearson correlation analysis, and the results are shown in Table 2.

### 3.5. Model of the Influence of Ecosystem Recreational Services on Visitor Well-Being

**Figure 5:**
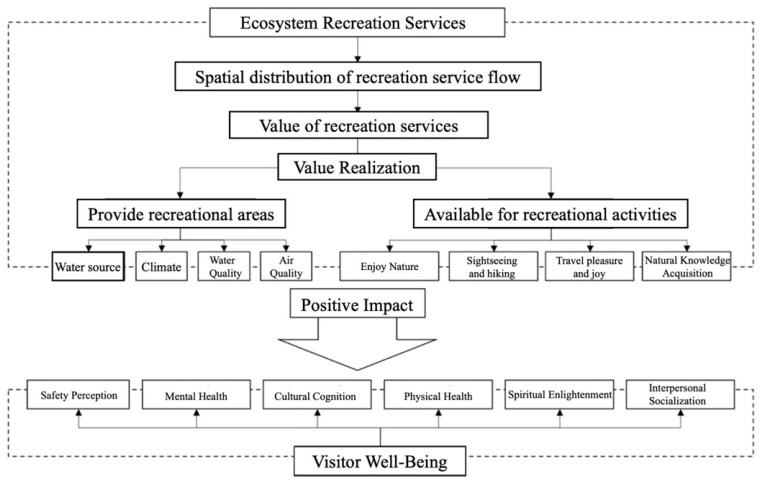
Model of the impact of ecosystem recreational services on the well-being of tourists.

Based on the above analysis, we constructed a model of the influence of ecosystem recreational services on visitor well-being in Qianjiangyuan National Park (Figure 5). The influence was concentrated in the fact that ecosystems provide recreational places and activities for tourists, and in particular, have a positive impact on the well-being of tourists, such as physical and mental health, interpersonal socialization and cultural cognition.

1. Ecosystem recreational services provide favorable recreational sites for tourists, such as Qianjiangyuan National Forest Park, Qixi Reservoir and Gutian Mountain, which are consistent with the spatial distribution of recreation potential and have a significant positive impact on tourists’ well-being [37]. The Qianjiangyuan ecosystem is an important water source in Zhejiang, growing a rich diversity of flora and fauna, which provides a pleasant natural environment with fresh air and clear water, and good perception of visitor safety. The Qiantang River is also the mother river of Zhejiang, which nurtures the local natural ecology and history and culture, and therefore has a more significant impact on the well-being of visitors’ cultural perception and spiritual enlightenment. In addition, the national park has a very high forest cover and a large area of subtropical broad-leaved forest, which helps regulate the climate, nourish water and purify the air, so the national park provides visitors with a recreational and health-care place with a good natural ecology suitable for recreational and health-care activities. The fresh air is conducive to rejuvenation, relaxation and disease prevention and provides significant improvement to the well-being of visitors’ mental and physical health.
2. Ecosystem recreational services provide conditions for visitors to engage in a variety of recreational activities, such as sightseeing and hiking, which have a significant positive impact on visitors’ well-being [38]. In the process of recreation, visitors learn about nature, history and culture, providing a pleasant and happy recreational experience and relieving anxiety and stress. Therefore, it has a significant positive impact on the well-being of tourists’ cultural awareness, physical health and mental health. On the other hand, unique natural landscape scenery can purify people’s spirits and minds, stimulate creativity and inspiration, and make people love nature more. Therefore, it has a significant positive impact on the well-being of visitors’ spiritual enlightenment.
3. The effect of recreational activities on the interpersonal social well-being of tourists is greater than that of recreational places. There is a significant positive correlation between recreational activities and tourists’ interpersonal social well-being, while the correlation is not significant between recreational places and social well-being. The dynamic interaction with the local environment and residents during the experience of recreational activities is conducive to increased intimacy among peers and the establishment of new social relationships with local residents or travel companions. Therefore, dynamic recreational activities are more significant in enhancing interpersonal social well-being compared to static recreational sites.

## 4. Conclusions

### 4.1. Spatial Distribution and Value Transformation of Ecosystem Recreational Services

This study identified the supply and demand of ecosystem services and the service flow paths between the two, including its recreation potential, recreation demand and the actual service flow between the two. It analyzed the spatial patterns and spatial relationships of their flows to achieve spatial trade-offs and synergies in the value of ecosystem services [39]. On this basis, this study used TCM to assess the value of ecosystem recreational services, translating the abstract service value into monetary value.

In terms of natural use, land types such as grassland, woodland and water have high potential for recreational services [40,41]. These land types are rich in natural resources, have a beautiful and pleasant environment, and are suitable for people to carry out a variety of leisure and recreational activities in them, such as hiking, walking and rafting. In contrast, the potential for recreational services on cultivated land and construction land is relatively low. Among them, arable land is mainly used for agricultural production, and it is difficult to carry out recreational activities in the arable land area. Construction land includes residential land, commercial land and transportation facilities, with heavy traces of artificial construction and a low potential for recreational services.

The actual flow of ecosystem recreational services in Qianjiangyuan National Park is about 12.1 persons/km^2^ per year, and each person can enjoy a recreational value of about 1456.62 RMB per trip on average. Due to the impact of the pandemic, this may change based on the accessibility of national parks and the costs people bear to visit. The distribution of areas with high recreational value is consistent with the spatial distribution of the actual flow of ecosystem recreational services, mainly concentrated in local areas and urban areas with a developed economy, high population density and high accessibility. In particular, there is a significant positive relationship between ecosystem recreational services and visitor well-being. The impact of ecosystem recreational services is mainly reflected in the recreational sites and recreational activities provided by ecosystems. Visitors’ perception of well-being in terms of physical and psychological health, safety perception and spiritual enlightenment is more significant.

### 4.2. Implementation of Ecosystem Recreational Services in Visitor Well-Being

The value of ecosystem services lies in how the biophysical attributes of ecosystem service functions are translated into ecosystem service potentials that must be mobilized to make these potentials provide ecosystem services [42]. The questionnaire study examined local perceptions of ecosystem recreational service functions. This study constructed a scale to measure visitor well-being based on ecosystem service functions. It analyzed the enhancement of local human well-being by the value of ecosystem recreational services, including psychological, physical, spiritual, cultural, social and safety aspects. The natural recreational resources and accessibility of national parks are closely linked to the well-being of visitors.

There is a significant positive correlation between national park ecosystem recreational services and six dimensions of visitor well-being. In terms of recreational sites, Qianjiangyuan, as the birthplace of Qiantang River, is an important water source in Zhejiang Province. Its ecosystem regulates the local climate, purifies the air and provides visitors with a superior environment for recreation. From the viewpoint of recreational activities, visitors can enjoy nature sightseeing, hiking and other recreational activities in the national park, feel pleasure and joy in the process of recreation, and gain knowledge of nature. In addition, the influence of recreational activities on the interpersonal social well-being of visitors is much greater than that of recreational sites, and people are more likely to establish emotional ties and enhance social relationships in the dynamic interaction of recreational activities.

### 4.3. Practical and Theoretical Implications

Currently, research on the valuation of cultural services of ecosystems is gradually changing to the measurement of the non-material benefits derived from ecosystem services. In addition to the indigenous people in the ecosystem, tourists as outsiders will also benefit from the ecosystem, and the well-being of tourists is also affected by ecosystem services. We constructed an integrated framework for assessing ecosystem services at the macro level versus exploring the impacts of well-being at the micro level, which provides new ideas for ecosystem assessment and application. The research framework is based on the ESTIMAP. Using RS image data and GIS, this study classified the land use types in the region and analyzed the natural resource profiles in the study area. Expert scoring was conducted based on its natural attributes, and it assessed the value of recreational functions for its ecosystem cultural services. It used an ecosystem service mapping tool to analyze the value of ecosystem recreational services in Qianjiangyuan National Park. This graphical research method and expression can fully and intuitively analyze the coverage and value of national park ecosystem services.

In the planning and management process of national parks, protected areas and urban parks, it is necessary to fully consider the demand of recreational services in the surrounding ecosystems, make rational use of natural resources and improve transportation accessibility. In this way, the potential of ecosystem services can be exerted, the value transformation of ecosystem recreational services can be realized, and the well-being of tourists can be improved. Studying the ecological system cultural service value of Qianjiangyuan National Park can provide experience for the protection and development of other national parks and similar subtropical ecosystems and has guiding significance for the development practice of national parks. Through the establishment and integration of substantive management institutions, Qianjiangyuan National Park can realize the unity, standardization and efficiency of natural resource protection and management in the national park pilot area. It forms experience that can be used for reference and promotion in the construction of national parks and provides innovative ideas for the construction of ecological civilization in the surrounding areas of Zhejiang Province and the source areas of rivers.

### 4.4. Limitations and Future Research

In the process of research, due to the limitation of objective resources such as time, materials and manpower, as well as subjective limitations such as personal ability, there are still some limitations in the research. First of all, this study used the questionnaire survey method to investigate the research site. The questionnaire design process involved ecosystem services and well-being. The quality of the questionnaire was very high. There are still some shortcomings. At the same time, during the research process, due to time and manpower constraints, the distribution of the questionnaire itself also had a certain randomness. Secondly, due to the impact of the COVID-19 epidemic, the tourism industry has been hit and still has not fully recovered. The number of tourists visiting Qianjiangyuan National Park is relatively small. During the questionnaire survey, the sample size was relatively small, and the provinces covered by the tourists surveyed were not complete. Moreover, considering the difference between the low and peak seasons of local tourism, many tourist sources are not included in the results, which has a certain impact on the evaluation of recreational value.

In the future, research can be conducted on the following aspects. Firstly, the value of ecosystem recreational services is not static; it will change dynamically at different times and under different environments. Under the influence of the epidemic, low accessibility and high visiting costs will reduce people’s perception of the value of recreational services. Future research can study the dynamic changes of the recreational service value of this ecosystem for a certain time span. Secondly, the research object of this paper is mainly tourists, and it mainly discusses the impact of ecosystem recreational services on tourists’ well-being. However, the objects of ecosystem services are not only tourists but also local residents. If the literature and survey data are easily available, and using reasonable and scientific statistical analysis methods, the impact of ecosystem services on the well-being of residents can be studied. Thirdly, future study should include other service functions of the ecosystem, such as service value and service flow such as supply services and regulation services, and comprehensively measure the ecosystem service value of Qianjiangyuan National Park, which is conducive to discussing the important value of the national park, including ecological, economic and social aspects. In the future development process, the ecosystem service radiation range will be more extensive.

## Figures and Tables

**Table 1:** The well-being scores of tourists in Qianjiangyuan National Park.

Well-Being Factor	No.	Title of Item	Min.	Max.	Average	Standard Deviation
Safety Perception	W1	The climate of Qianjiangyuan is mild and suitable for human habitation	1	5	4.66	0.645
	W2	The security of Qianjiangyuan is good and the environment is safe	1	5	4.63	0.655
	W3	The air in Qianjiangyuan is clean and pollution-free	1	5	4.75	0.557
	W4	The spring water in Qianjiangyuan is clear and of good quality	1	5	4.75	0.585
	W5	The fruits and vegetables in Qianjiangyuan are fresh and pollution-free	1	5	4.49	0.798
	W6	This place has increased my intimacy with my family and friends	1	5	4.44	0.836
Mental Health	W7	I feel relaxed in Qianjiangyuan	2	5	4.53	0.690
	W8	I feel inner peace at Qianjiang source	2	5	4.46	0.745
	W9	My anxiety and stress are relieved by the Qianjiangyuan tour	1	5	4.39	0.753
	W10	I feel happy and joyful at Qianjiangyuan	2	5	4.58	0.680
	W11	Enjoying the natural scenery can rejuvenate me	1	5	4.52	0.731
Cultural Cognition	W12	I learned about the history and culture of this place	1	5	3.84	1.011
	W13	I learned about geography and geology	1	5	3.87	1.002
	W14	I gained some knowledge of flora and fauna	1	5	4.05	1.020
	W15	Qianjiangyuan has enhanced my understanding of the local terroir	1	5	4.11	0.983
Physical Health	W16	Fresh air nourishes the lungs and prevents respiratory diseases	1	5	4.57	0.692
	W17	Hiking here can strengthen my body	2	5	4.59	0.668
	W18	Hiking and relaxing are good for my mood and blood pressure	1	5	4.46	0.730
Spiritual Enlightenment	W19	Natural scenery inspires my creativity and inspiration	1	5	4.13	0.981
	W20	Natural landscape can purify the spirit and mind	1	5	4.45	0.823
	W21	Mountain climbing strengthens my will and makes me more confident	1	5	4.13	1.038
	W22	This place makes me feel poetic and love nature	1	5	4.59	0.710
Interpersonal Socialization	W23	The people are friendly and I got to know the locals	1	5	3.79	1.250
	W24	I also made new friends during the trip	1	5	3.77	1.228

**Table 2:** Correlation analysis of tourist’s well-being, reflecting the value of recreational services.

Recreational Services Value	Specific Indicators		Safety Perception	Mental Health	Cultural Cognition	Physical Health	Spiritual Enlightenment	Interpersonal Socialization
Recreational areas	Water source	Pearson Correlation	0.430 **	0.334 **	0.242 **	0.332 **	0.294 **	0.152 *
		Sig.	0.000	0.000	0.000	0.000	0.000	0.012
	Climate	Pearson Correlation	0.521 **	0.437 **	0.313 **	0.425 **	0.318 **	0.102
		Sig.	0.000	0.000	0.000	0.000	0.000	0.092
	Water quality	Pearson Correlation	0.518 **	0.445 **	0.239 **	0.484 **	0.314 **	0.075
		Sig.	0.000	0.000	0.000	0.000	0.000	0.215
	Forests	Pearson Correlation	0.462 **	0.395 **	0.210 **	0.414 **	0.275 **	0.083
		Sig.	0.000	0.000	0.000	0.000	0.000	0.168
Available for recreational activities	Sightseeing and hiking	Pearson Correlation	0.497 **	0.386 **	0.232 **	0.391 **	0.313 **	0.137 *
		Sig.	0.000	0.000	0.000	0.000	0.000	0.023
	Travel pleasure	Pearson Correlation	0.487 **	0.487 **	0.230 **	0.393 **	0.360 **	0.151 *
		Sig.	0.000	0.000	0.000	0.000	0.000	0.012
	Nature Knowledge	Pearson Correlation	0.421 **	0.488 **	0.402 **	0.397 **	0.444 **	0.390 **
		Sig.	0.000	0.000	0.000	0.000	0.000	0.000

** Significantly correlated at the 0.01 level. * Significantly correlated at the 0.05 level.

**Figure A1:**
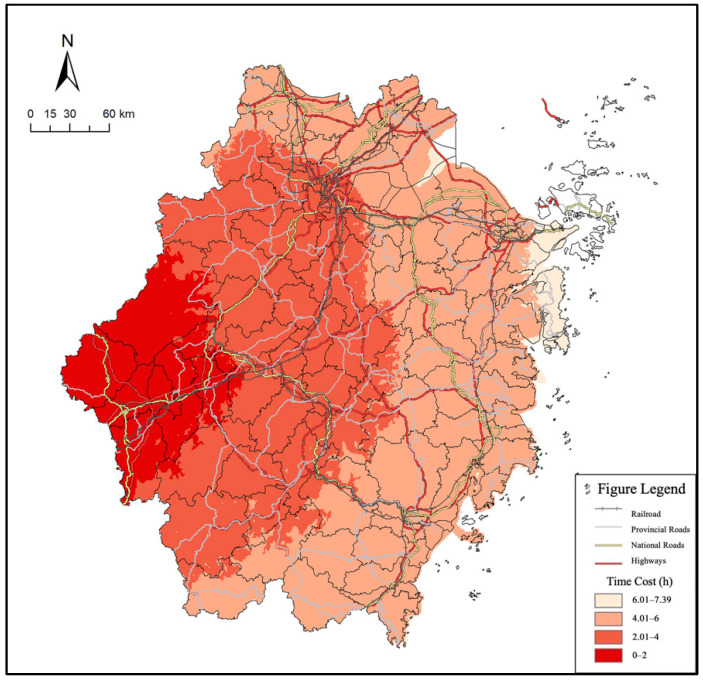
Time cost of reaching Qianjiangyuan National Park in Zhejiang Province.

## Data Availability

The data presented in this study are available on request from the corresponding author. The data are not publicly available due to privacy restrictions.

## Appendix A

**Table A1:** Recreation potential scores of land types in Qianjiangyuan National Park.

Land Use Types	Recreation Potential Score
Cropland	4.24
Tree woodland	7.29
Bamboo forest	7
Shrubland	6.53
Orchard	6.71
Grassland	8
Rivers and lakes	7.76
Reservoir pond	5.65
Continuous large area of residential land	4.06
Discontinuous dotted commercial land	4.59
Roads and transportation facilities	3.76
